# Unilateral bullous exudative retinal detachment in Behçet's disease

**DOI:** 10.11604/pamj.2014.18.127.3095

**Published:** 2014-06-10

**Authors:** Zouheir Hafidi, Rajae Daoudi

**Affiliations:** 1 Université Mohammed V Souissi, Service d'Ophtalmologie A de l'hôpital des Spécialités, Centre Hospitalier Universitaire, Rabat, Maroc

**Keywords:** Retinal detachment, Behçet's disease, retinal vasculitis

## Image in medicine

We report an unusual case of Behçet disease presenting with retinal vasculitis and unilateral exudative retinal detachment. A 24 years old man presented with 1 week history of acute vision loss of both eyes. He reported a 3 years history of recurrent oral and genital ulcerations, with arthralgia of wrists and ankles. The best correct visual acuity was 6/30 in the right eye and 6/24 in the left eye. Slit lamp examination showed bilateral mild inflammation in the anterior chamber and vitreous. Funduscopy revealed bilateral extensive perivascular sheathing with scattered yellowish retinal infiltrates and hemorrhages. In the right eye there was, in addition, a bullous exudative retinal detachment involving the superior and inferior quadrants of the temporal retina (a, white arrows). Fluoresce in angiography (b) showed late staining of the retinal vasculature with diffuse dye leakage in both eyes. In the right eye, late frames showed an evident fluorescein pooling in the subretinal space (black arrows). The patient received intravenous bolus of methylprednisolone (1 g daily / 3days) leading to prompt clinical improvement. Azathioprine was also started at a dose of 150 mg daily. Exudative retinal detachment is an uncommon finding in Behçet's disease, and only few cases were reported in the medical literature. This entity could reflect the severity of the underlying disease. In addition the early set of the disease and male gender are usually associated with poor long term prognosis. This justifies our choice of giving azathioprine as an immunosuppressive therapy.

**Figure 1:**
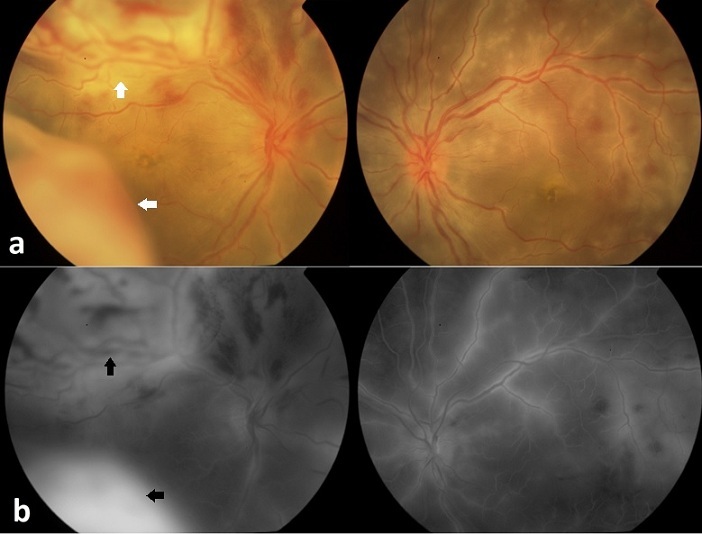
Funduscopy and fluoresce in angiography: (a) Extensive perivascular sheathing with scattered yellowish retinal infiltrates and hemorrhages in both eyes, with a bullous exudative retinal detachment involving the superior and inferior quadrants of the temporal retina in the right eye (white arrows). (b) Fluoresce in angiography showing late staining of the retinal vasculature with diffuse dye leakage in both eyes and fluorescein pooling in the subretinal space in the right eye (black arrows)

